# Short-term results of intra-articular injections of stromal vascular fraction for early knee osteoarthritis

**DOI:** 10.1186/s13018-022-03196-0

**Published:** 2022-06-11

**Authors:** Cristian Aletto, Lorenzo Giordano, Marco Quaranta, Arnaldo Zara, Donato Notarfrancesco, Nicola Maffulli

**Affiliations:** 1 Department of Musculoskeletal Disorders, Faculty of Medicine and Surgery, University of Salerno, 84084 Baronissi, Italy; 2 Clinica Ortopedica, Ospedale San Giovanni di Dio e Ruggi D’Aragona, 84131 Salerno, Italy; 3 Casa di Cura Salus, 84091 Battipaglia, SA Italy; 4 Barts and the London School of Medicine and Dentistry, Centre for Sports and Exercise Medicine, Mile End Hospital, Queen Mary University of London, 275 Bancroft Road, London, E1 4DG England; 5 Guy Hilton Research Centre, School of Pharmacy and Bioengineering, Faculty of Medicine, Keele University, Thornburrow Drive, Hartshill, Stoke-on-Trent, ST4 7QB England

**Keywords:** MSC, ADSC, SVF, Knee, Osteoarthritis

## Abstract

**Background:**

In knee osteoarthritis, progressive degeneration of the articular cartilage surface produces disability and chronic pain. Intra-articular injections of stromal vascular fraction (SVF) could be an innovative approach to manage patients with early knee osteoarthritis.

**Methods:**

Between June 2019 and November 2020, 123 patients were recruited to receive intra-articular injection of SVF. Radiographic evidence of degenerative joint disease was classified according to Kellgren and Lawrence grades. Knee injury and osteoarthritis outcome score (KOOS) and visual analog scale (VAS) were collected preoperatively, at 1 month, and after 6 months from injection.

**Results:**

There was a statistically significant improvement of KOOS and VAS of all patients to 6 months (*p* < 0.05). The mean KOOS before injection was 51.4 ± 16.5, after 1 month it was 75.5 ± 15.8, and at 6 months it was 87.6 ± 7.7. Stratifying the mean KOOS according to Kellgren–Lawrence Grades, the difference remained statistically significant (*p* < 0.05). The patients’ mean VAS before injection was 6.5, after 1 month it was 3.5, and after 6 months it was 2.4. No complications were observed.

**Conclusions:**

Intra-articular knee injection of SVF is safe and effective to ameliorate the clinical and functional scores in patients with early knee osteoarthritis for 6 months**.**

## Background

The knee is the weight-bearing joint most frequently plagued by osteoarthritis (OA). Conservative management of OA includes physical exercises, body weight reduction, drugs, hyaluronic acid injections with or without corticosteroid, and platelet-rich plasma (PRP) injection. Nonsteroidal anti-inflammatory drugs (NSAIDs) provide short-term benefits, while corticosteroids may worsen the clinical picture in the long term [1, 2]. Hyaluronic acid (HA) injections are a repeatable option which may reduce pain, but HA does not reverse or repair the damaged cartilage [3]. Effectiveness of microfractures (MF), autologous chondrocyte implantation (ACI) or matrix-applied chondrocyte implantation (MACI), and autologous chondrocyte transplantation (ACT) in the management of OA is uncertain and unpredictable, suggesting benefits especially in the treatment of focal chondropathies [4–6]. In younger patients with medial compartmental osteoarthritis of the knee, valgus high tibial osteotomy reduces pain and improves knee function [7]. Definitive solution to provide pain relief for the latest stages of OA is joint replacement, but the limited longevity of prostheses may restrict their use in younger and active patients [8]. Therefore, the ideal treatment for OA should restore the biomechanical and biochemical properties of damaged cartilage focusing on cartilage repair and restoration [9]. This may be possible in early knee OA (Kellgren–Lawrence grade 1–2), while, in the more advanced stages of knee OA, to reverse the progression of this condition is problematic [10].

Intra-articular cell therapies have recently emerged as a method to manage the early stages of knee osteoarthritis (KOA). Mesenchymal stem cells (MSCs) can be considered a potential biological approach to articular cartilage restoration given their properties of multi-lineage differentiation potential, self-renewal, and immunomodulatory capacity [11]. MSCs may be able to produce new cartilage, releasing factors that stimulate cartilage formation by resident chondrocytes or other cells in the joint, and inhibit joint inflammation [12]. Indeed, most of the effects of these cells are consequent to their paracrine effect rather than their potential differentiation into chondrocytes [13]. MSCs release growth factors and anti-inflammatory cytokines which inhibit apoptosis, stimulate endogenous cell proliferation and repair, and stimulate angiogenesis and vessel stability, improving blood flow in the affected joint by contributing to endogenous tissue repair [7]. Although joint fluid contains MSCs, only a limited number of them can differentiate into chondrocytes, so administration of exogenous MSCs can improve articular cartilage repair [11].

MSCs can be collected from different tissue sources. Autologous cells induce no rejection, carry no risk of disease transmission, and are less tumorigenic than embryonic stem cells [14]. Bone marrow and adipose tissue are the main sources of MSCs: they can be harvested in a minimally invasive fashion and can be minimally manipulated intra-operatively with sterile devices [15]. In particular, adipose/fat tissue contains a greater concentration of MSCs than bone marrow (0.01–0.1% vs. 0.001–0.01%), it provides an abundant source of stromal vascular fraction (SVF) cells for immediate administration and, through enzymatic digestion, it can also give rise to a substantial number of multipotent adipose-derived stromal cells (ADSCs) [6, 16]. These cells of the SVF have the potential to differentiate into adipogenic, osteogenic, chondrogenic, and other mesenchymal lineages [17]. Intra-articular injection of autologous micro-fragmented adipose tissue in patients with KOA increased glycosaminoglycan content in hyaline cartilage [18]. ADSCs are obtained by mechanical or enzymatic treatment which involve many processing steps, high economic burden, and restrictions associated with cell expansion and extensive manipulation [18]. Unlike ADSC, SVF can be readily obtained from lipoaspirate without the need for cell separation or culturing, which makes SVF more cost-efficient and convenient [19]. The use of SVF has shown good results in all stages of knee OA, and in various age groups, their safety and efficacy significantly increased clinical and imaging outcomes [20, 21]. Therefore, concomitant strategies could be implemented in conjunction with SVF injection treatments: high tibial osteotomy, platelet-rich plasma, and hyaluronic acid injections [22].

Although MSCs have shown efficacy in clinical studies, evidence on their efficacy remains unclear in KOA, given cell heterogeneity and concomitant procedures.

This study analyses the short-term clinical and functional results of patients with early KOA treated with one single intra-articular injection of adipose-derived stem cells without expansion or enzymatic treatment.

## Methods

### Study design

Between June 2019 and November 2020, 123 patients undergoing intra-articular injection of adipose stem cells were recruited. The patients’ age, sex, body mass index (BMI), previous surgery on the affected knee, and medical comorbidities were recorded at pre-operative assessment. Selection criteria were clinical findings of KOA with radiographic evidence of degenerative joint disease on standing anteroposterior and lateral radiographies. Exclusion criteria were age over 75 years, body mass index < 18 or ≥ 30, patients with a severe (> 10°) varus or valgus deformity, severe Kellgren–Lawrence grade (IV) on radiographs, recent trauma with acute involvement of ligaments and/or menisci, and infectious or inflammatory joint disease. Patients who received both MSCs harvesting and knee arthroscopy at the same time were excluded. The Kellgren and Lawrence classification was used to grade the severity of osteoarthritis: grade 0 is absence of radiographic signs of osteoarthritis; grade 1 is characterized by doubtful joint space narrowing and possible osteophytic lipping; in grade 2, there are osteophytes and possible joint space narrowing; grade 3 is defined by moderate multiple osteophytes, definite narrowing of joint space and some sclerosis and possible deformity of bone ends; grade 4 is a severe condition with large osteophytes, marked joint space narrowing, severe sclerosis, and definite deformity of bone ends. The subcutaneous adipose tissue on the abdomen was evaluated at the first consultation because the “relative” absence of fat and the presence of abdominal scars could complicate the harvesting. Medical history, Knee Injury and Osteoarthritis Outcome Score (KOOS), and visual analog scale (VAS) were collected by two orthopedic surgeons (CA and LG) for all patients before lipoaspiration and at the first and sixth month from injection. The Knee Injury and Osteoarthritis Outcome Score (KOOS) evaluates the course of knee injury and treatment outcomes. It assesses 42 items in 5 separately scored subscales: Pain (nine items), Symptoms (seven items), Function in daily living (ADL) (17 items), Sport and Recreation Function (five items) and Quality of Life (QOL) (four items). Scores are transformed to a 0–100 scale, with zero representing extreme knee problems and 100 representing no knee problems. The visual analog scale (VAS) is used to classify knee pain; it ranges from no pain (0) to an extreme amount of pain (10). Informed consent was obtained from all individual participants included in the study.

### Lipoaspirate harvesting

**Fig. 1:**
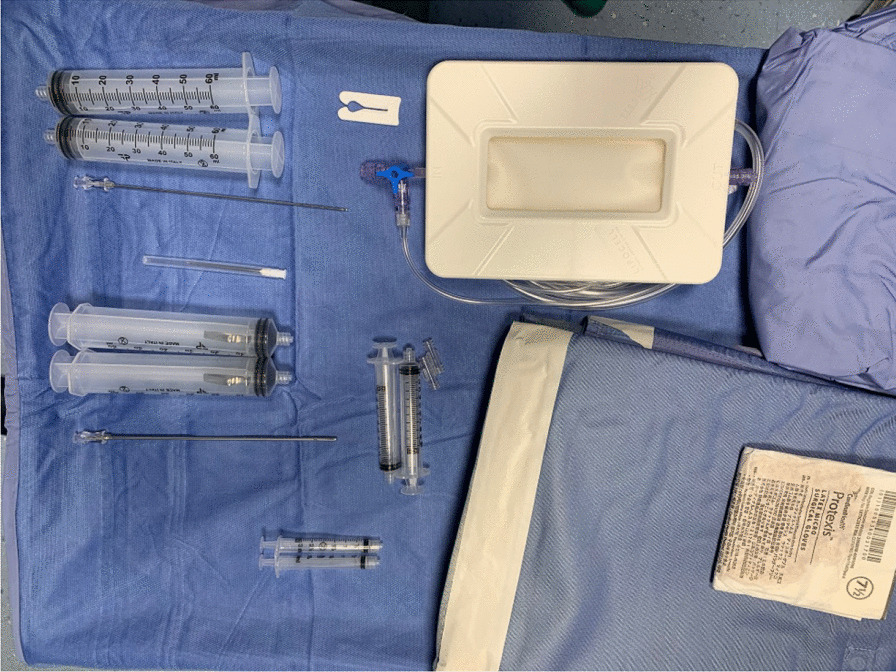
Lipocell kit

**Fig. 2:**
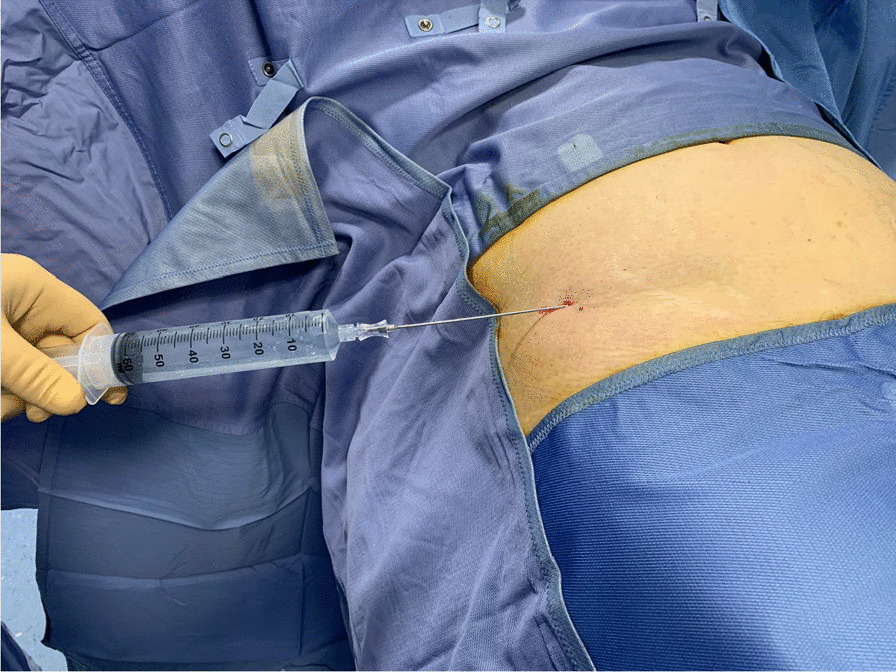
Klein solution injection

Patients were placed supine; the abdomen was prepared in a standard fashion with betadine and chlorhexidine. The surgical field was prepared, and 1 ml of Lidocaine 2% was injected at the site of skin incision. All procedures were performed by two fully trained surgeons (DN and AZ). The preferable harvesting area is the iliac and lumbar abdominal area or the periumbilical area. After performing a small skin incision, an infiltration fenestrated blunt cannula (16G) connected to 50 ml syringes was used to inject Klein solution homogenously. This solution contains 250 ml of NaCl 0.9%, 20 ml of Lidocaine 2%, and 0.5 ml of Epinephrine 1 mg/ml. Transverse movements with the cannulas should be avoided, and the solution should be injected during retrograde movements of the cannula. The distribution of 150–200 ml of the Klein solution in the subcutaneous layers is facilitated by digital manipulation of the abdomen (Figs. 1 and 2).

**Fig. 3:**
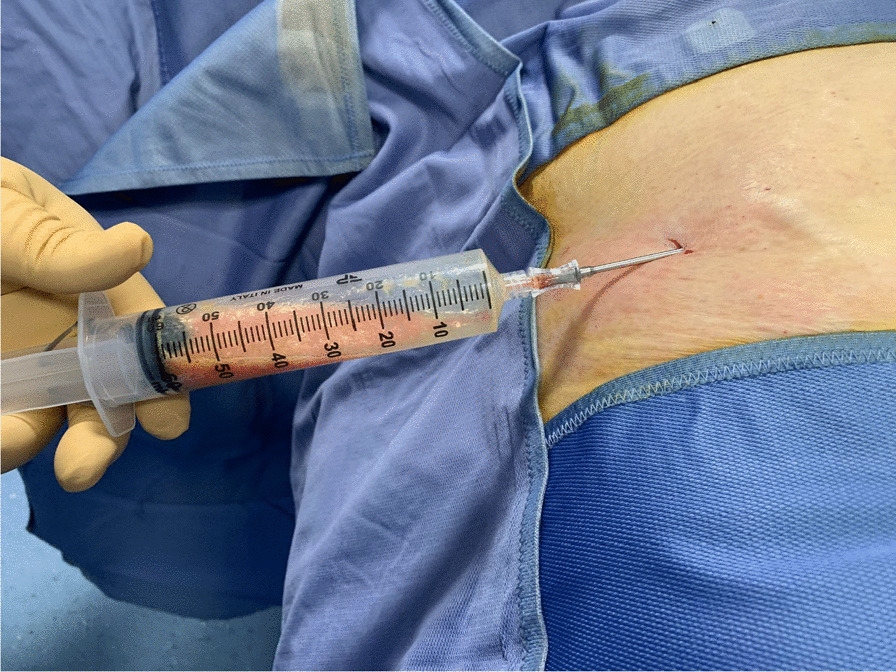
Harvesting

**Fig. 4:**
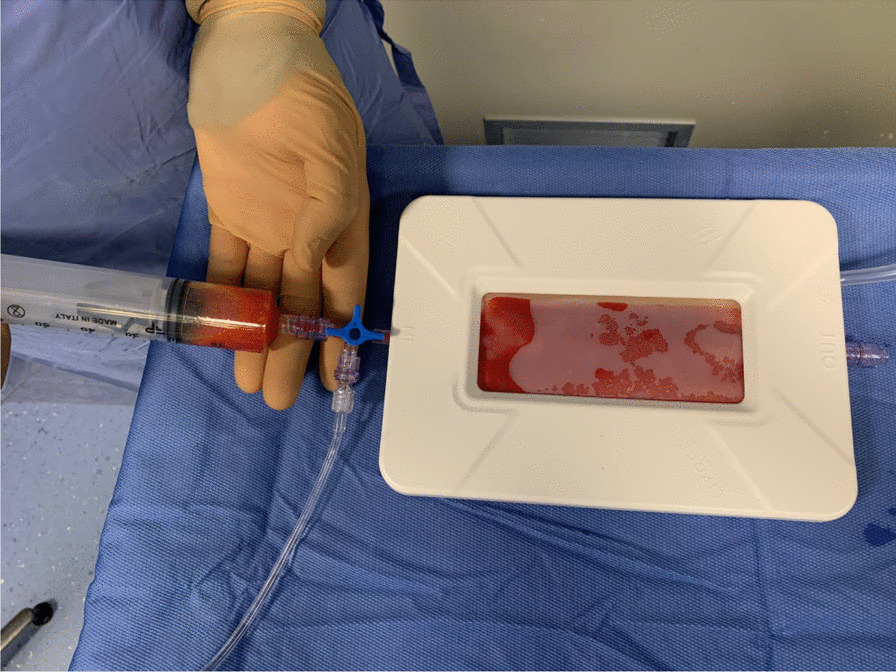
Lipoaspirate harvested is transferred into lipocell device

**Fig. 5:**
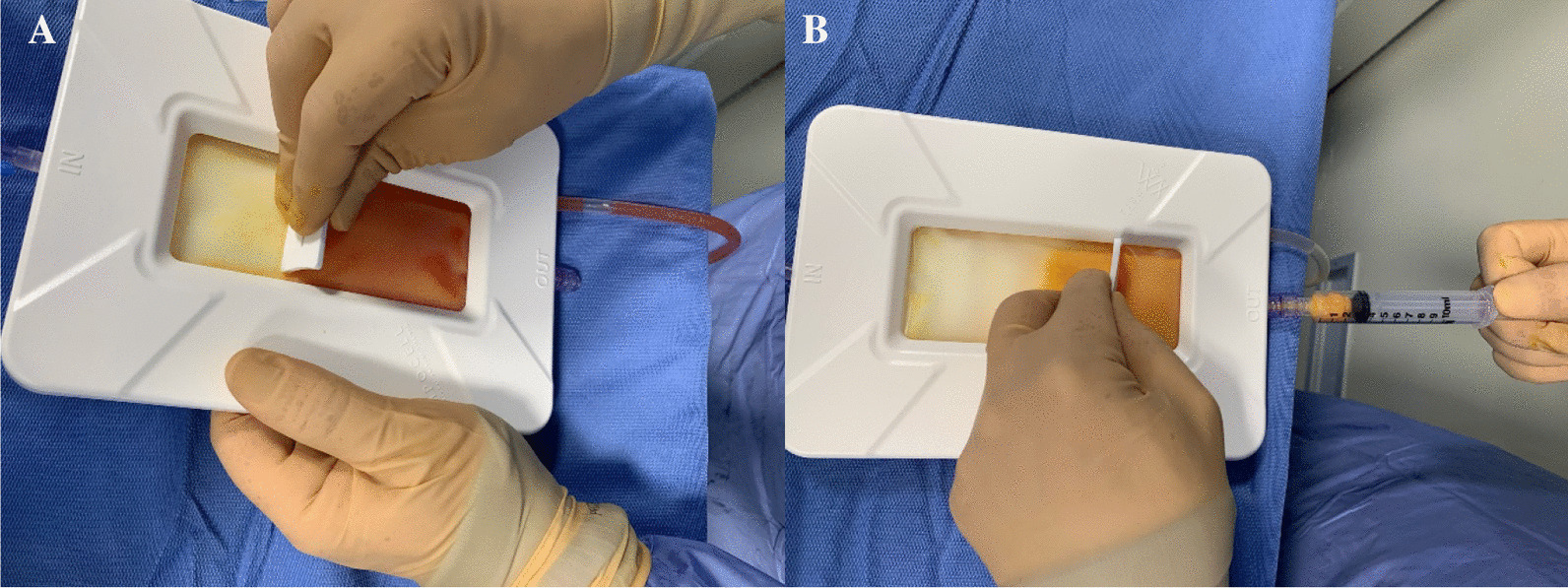
Lipoaspirate processing (**A**), Purified lipoaspirate (**B**)

After 10 min, an aspiration cannula (13G) connected to a self-blocking syringe is introduced in the subcutaneous fat and lipoaspiration can start. The block system produces negative pressure inside the syringe, allowing to harvest the lipoaspirate from the previously infiltrated areas just moving the plunger back (Figs. 3, 4 and 5).

**Fig. 6:**
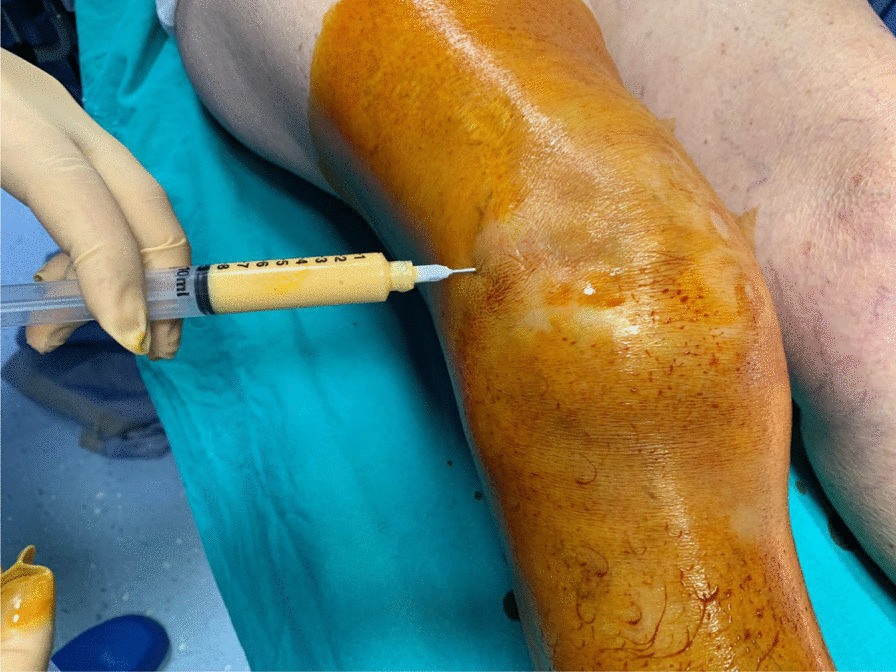
Superolateral injection

After obtaining the lipoaspirate, a compressive dressing is applied. 60–90 ml of lipoaspirated fat is collected from the abdominal subcutaneous fat and processed using the Lipocell system (Tiss’You, RSM). The lipoaspirate is transferred into the Lipocell device where it is dialyzed with a filter and washed with 500 ml of Ringer lactate or NaCl 0.9%. When the lipoaspirate becomes yellow and the outflow irrigation is almost clear of blood, it is ready to be collected using a 10-ml syringe and to be injected (10–15 ml) intra-articularly. The choice of injection portal may be either superolateral under the patella with the knee extended or through the inferomedial or inferolateral soft part of the knee with the knee flexed to 90°. After the injection, the knee is flexed and extended several times to diffuse the product in the joint (Fig. 6).

At discharge, all patients were instructed to wear an elastic dressing for a week to reduce the occurrence of hematoma on the abdomen. Patients are fully weight bearing with crutches, and full unaided weight bearing on the treated knee is allowed after 1 week. All patients were instructed to perform isometric quadriceps exercises and started physiotherapy after 1 week.

### Statistical analysis

The Student *t* test was used to compare the means of KOOS and VAS values before and after surgery. Statistical significance was set at *p* < 0.05.

## Results

**Fig. 7:**
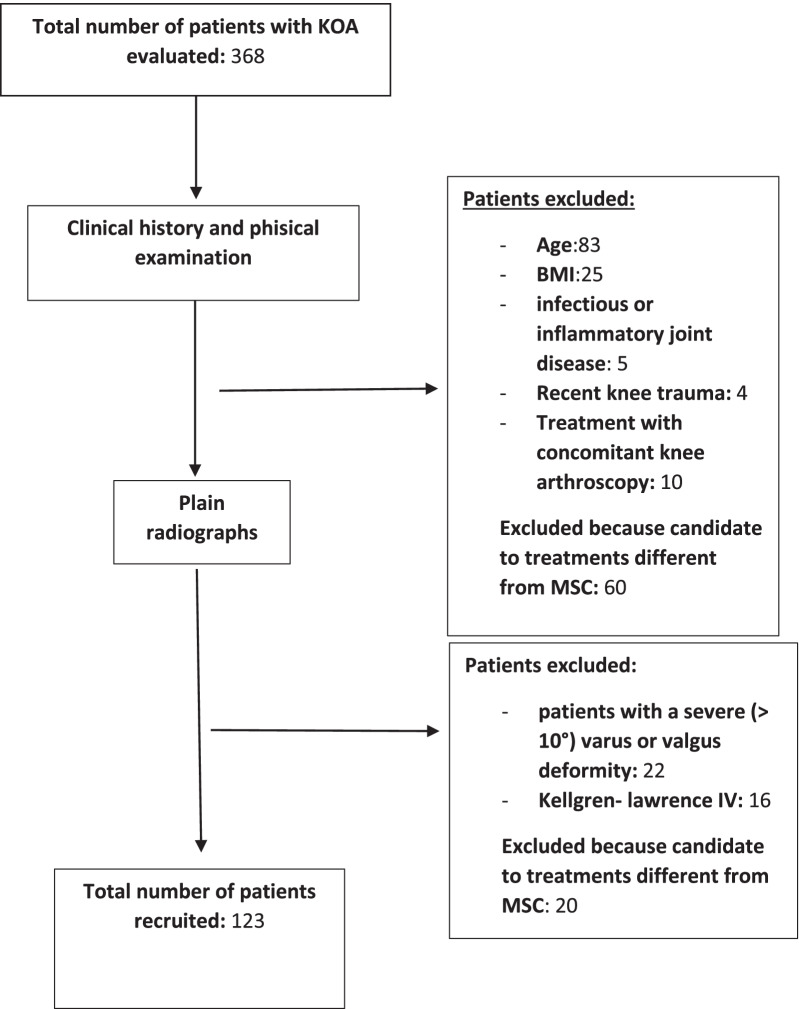
STROBE diagram

A total of 123 patients satisfied the inclusion criteria and underwent injection of adipose stem cells, with a mean age of 57 years. There were more females (*n* = 66, 53.4%) than males (*n* = 57, 46.4%) with an average body mass index (BMI) of 27. The STROBE diagram shows the various phases of the study (Fig. 7).

Regarding previous treatments, 18 patients (14%) had medial meniscus surgery, 4 patients (3%) had undergone anterior cruciate ligament reconstruction, 32 patients (26%) had tried hyaluronic acid injections, 2 patients (1%) had undergone tibial osteotomy, and 67 patients (54%) had not received other treatments before the index procedure (Table 1).

**Table 1:** Patients characteristics (no:123)

	*N*°/%	Mean value
Sex	M: 57 (46.4%)	
	F: 66 (53.4%)	
Age (years)		57
BMI (kg/m^2^)		27
Medial meniscus surgery	18 (14%)	
Anterior Cruciate Ligament reconstruction	4 (3%)	
Tibial osteotomy	2 (1%)	
HA injections	32 (26%)	
No other treatments before	67 (54%)	

Radiographic signs of grade I of Kellgren–Lawrence were evident in 52 patients (42%), 61 patients (49%) showed grade II, and 10 patients (8%) showed grade III. The mean KOOS of all patients before injection was 51.4 ± 16.5, at the first month from injection it was 75.5 ± 15.8, and at the sixth month from injection it was 87.6 ± 7.7 (*p* < 0.05). The Student’s *t* test showed statistically significant difference between the mean values of KOOS before and at 1 and 6 months after injection (*p* < 0.05). Stratifying mean KOOS on Kellgren–Lawrence Grades, the difference between values remains statistically significant (*p* < 0.05) (Table 2).

**Table 2:** Mean KOOS before and after SVF injections stratified by Kellgren-Lawrence grades

Kellgren–Lawrence	*N*°/%	Mean KOOS before	Mean KOOS (1st month)	Mean KOOS (6th month)
I	52 (42%)	55.37 ± 16.9	79.67 ± 9.7	89.82 ± 5.8
II	61 (49%)	49.9 ± 15.4	75.6 ± 10.8	85.9 ± 8.5
III	10 (8%)	45 ± 15	75.6 ± 6.1	84.1 ± 10.7

As some patients had undergone previous treatments before adipose stem cell injections, we stratified the patients in two groups based on previous treatments on the affected knee. “Naïve” patients (i.e., patients which did not receive previous treatments on affected knee) were 67, while “not-naïve” patients were 56. Table 3 shows KOOS before and after treatments for the two groups.

**Table 3:** Mean KOOS before and after SVF injections basing on previous treatment on affected knee

	*N*°/%	Mean KOOS before	Mean KOOS (1st month)	Mean KOOS (6th month)
Naïve knees	67 (54%)	53.4 ± 17.7	73.7 ± 19.2	86.9 ± 7.2
Not Naïve knees	56 (46%)	48.9 ± 14.9	77.8 ± 10.2	88.7 ± 8.4

The Student’s *t* test showed statistically significant difference between the mean values of KOOS for naïve patients before and at 1 and 6 months after injection (*p* < 0.05). The same results were obtained with not naïve patients.

The patients’ mean VAS before injection was 6.5, after 1 month it was 3.5, and after 6 months it was 2.4. The Student’s *t* test showed statistically significant difference between the mean values of VAS before and at 1 and 6 months after injection (*p* < 0.05). No complications were observed in the patients treated.

## Discussion

Recently, clinicians and researchers are focusing on prevention of progression of KOA through the use of mesenchymal stromal stem cells (MSCs). These cells, with their chondrogenic and anti-inflammatory properties, may well reverse the first stages of KOA, reducing synovitis, cartilage degeneration, and osteophyte formation [23]. Adipose tissue and bone marrow are sources of MSCs, and adipose tissue-derived MSCs are easy to collect for clinical application. Adipose tissue is ubiquitously available and is easily accessible in large quantities with minimally invasive harvesting procedures [15]. In this study, we assessed the short-term clinical outcome of 123 patients with stage I, II, and III knee OA treated with intra-articular injection of SVF obtained from their own abdominal subcutaneous fat. Our results show a statistically significant improvement in terms of KOOS and VAS for up to 6 months. One single injection of lipoaspirate reduces knee pain and improves function after 1 month from the injection, and the benefits are maintained for 6 months independently of the Kellgren–Lawrence grades before injection. These results are in accordance with other similar studies. For example, Castellarin et al. [4] reported good functional and clinical results after 1 year on 92 patients using the same harvesting device. Adriani et al. [24] reviewed 30 patients who received an autologous percutaneous fat injection for the treatment of KOA. All patients reported improvements in terms of VAS and WOMAC, but a slight deterioration was evidenced at 1 year. Lee et al. [25] conducted a prospective double-blinded, randomized controlled, clinical trial; they compared 12 patients injected with ADSC from abdominal subcutaneous fat with 12 knees injected with normal saline. A single injection of adipose-derived mesenchymal stem cells led to a significant improvement of the WOMAC score at 6 months, while the control group showed no significant changes in WOMAC score at 6 months. On the other hand, considering MRI, there was no significant change in cartilage appearance at 6 months in the ADSC group, whereas the defect in the control group was increased. Some authors [17, 26] have used adipose stem cells in grades III and IV of Kellgren–Lawrence using three consecutive escalating doses of ADSCs, with improvement in clinical, functional and VAS scores. In our relatively young patients with low grades (I–III) of KOA, intra-articular SVF injections resulted in favorable clinical outcomes; we preferred not to treat with mesenchymal cell injection patients with Grade IV of Kellgren–Lawrence, as joint replacement is indicated in such patients. Considering the above studies, patients of appropriate age, BMI, and comorbidities, who prefer not to undergo surgery, could be ideal candidate for escalating doses therapy with adipose-derived stem cells injections.

MSCs may have limited clinical efficacy in high grade KOA, and more studies with high-level evidence [7] recommending the use of concomitant treatment including scaffolds, PRP or growth factors in any combination are needed [18, 27, 28]. Schiavone Panni et al. [29] proposed the concomitant use of adipose stem cells with knee arthroscopy debridement with a significant improvement in clinical and functional scores in patients with early KOA for 6–24 months. The present study excluded patients who underwent concomitant arthroscopy or other procedures, to try and focus on the actual effectiveness of adipose stem cells injection alone. In our patients, adipose stem cells alone give short-term pain relief in patients with early KOA (grade I, II and III).

The area of fat harvesting and patient age may influence the stem cell yield [30]; further studies would be necessary to identify the optimal cell concentration and environment needed for clinical application of intra-articular injection in knee OA [6, 31]. In one retrospective study conducted by Yokota et al. [32], isolated ADSCs were compared to SVF (59 vs. 69 knees) and no significant differences in clinical outcomes considering the Outcome Measures in Rheumatology-Osteoarthritis Research Society International (OMERACT-OARSI) criteria were observed between the two groups. On the other hand, there was a greater frequency of knee swelling in the SVF group maybe attributable to the presence of white blood cells in the heterogeneous SVF cell population. However, unlike ADSC, SVF is promptly accessible from lipoaspirate without separation and cell culturing, is cheaper and faster and the injection can be performed on the same day of the surgical procedure [6].

Autologous adipose stem cells show beneficial effects when compared to other MSCs to relieve pain and improve function, possibly because of the visco-supplementation effect of fat tissue on cartilage surfaces [33]. The most promising aspect of SVF is that adipose tissue is distributed all over the body, and with only 60–90 ml and a simple manipulation without expansion or enzymatic treatment we can obtain clinically relevant short-term results.

This study did not evidence any adverse events. Therefore, intra-articular administration of adipose tissue derivatives can be considered safe and less invasive than other approaches. No patient needed any additional surgical treatment within the follow-up period, and a second injection of adipose stem cells might be possible when the improvement of the first injection becomes ineffective. Biological treatments are obviously more expensive than other conservative treatment, but traditional treatments are often only palliative. Biological treatments offer the opportunity to regenerate tissues and delay the progression of KOA [34]. To date, injections of autologous purified adipose stem cells ensure only short-term results, while long-term results can be obtained with joint replacements. With the use of autologous SVF, a young and active patient can delay or even avoid major surgery such as total/partial knee arthroplasty.

The principal limitation of this study was that the potential biological effects of the adipose stem cells on the joint surface were not evaluated through MRI or arthroscopy, and we can only base our considerations on patients’ symptoms. Furthermore, there was no comparison with other conservative therapies; to assess the efficacy of adipose stem cells, a control group treated only with NSAIDS, or PRP, or HA or other conservative modalities would be needed.

Another important limitation of this study was that the follow-up (6 months) was short, but we point out that we wanted only to assess the short-term efficacy, and long-term efficacy will be the object of future studies.

## Conclusion

Isolated intra-articular knee injection of SVF is safe and effective to ameliorate the clinical and functional scores in patients with early KOA for 6 months. It can be considered an innovative approach able to prevent OA progression and delay total knee arthroplasty.

## Data Availability

Not applicable.

## Abbreviations

ACI: Autologous chondrocyte implantation
ACT: Autologous chondrocyte transplantation
ADL: Activity daily living
ADSCs: Adipose-derived stromal cells
BMI: Body mass index
HA: Hyaluronic acid
KOA: Knee osteoarthritis
KOOH: Knee osteoarthritis outcome score
MACI: Matrix-applied chondrocyte implantation
MF: Microfractures
MSCs: Mesenchymal stem cells
NSAIDs: Nonsteroidal anti-inflammatory drugs
OA: Osteoarthritis
PRP: Platelet-rich plasma
QOL: Quality of life
SVF: Stromal vascular fraction
VAS: Visual analog scale
